# Therapeutic potential of ultrasound for spinal cord injury

**DOI:** 10.4103/NRR.NRR-D-25-00978

**Published:** 2025-11-25

**Authors:** Ederlyn M. Perolina, Brad J. Raos, Maria Asplund, Darren M. Svirskis, Sachin S. Thakur

**Affiliations:** 1 Cure Programme, University of Auckland, Auckland, New Zealand; 2 School of Pharmacy, University of Auckland, Auckland, New Zealand; 3 Department of Microtechnology and Nanoscience, Chalmers University of Technology, Gothenburg, Sweden

**Keywords:** drug delivery, neuromodulation, neuronal regeneration, spinal cord injury, ultrasound

## Abstract

Spinal cord injury is a debilitating condition that often results in permanent impairments in motor, sensory, and autonomic functions. The potential for treating spinal cord injury with ultrasound is increasingly being recognized. Ultrasound addresses several key aspects of spinal cord injury, including promoting nerve healing by creating a regenerative environment, attenuating inflammation, and managing secondary complications. While preclinical studies show encouraging results, further translational efforts are necessary to adapt these therapies for clinical use. Insights gained from transcranial ultrasound applications, such as transducer selection, anatomical considerations, and pre-treatment planning, can provide valuable guidance for optimizing ultrasound treatments for spinal cord injury. This literature review highlights the exciting potential of ultrasound therapy in spinal cord injury research and the importance of refining treatment parameters and strategies for successful clinical translation.

## Introduction

Spinal cord injury (SCI) is a debilitating condition that affects motor, sensory, and autonomic functions, significantly impacting the quality of life of those afflicted. SCI begins with primary injury, where direct neuronal damage occurs (Ahuja et al., 2017). This initial damage then leads to secondary injury, which exacerbates the harm through processes such as reactive oxygen species production, increased inflammation, neuronal death, and the formation of an astro-glial scar, which acts as a physical and chemical barrier to regeneration (Anjum et al., 2020). This creates a significant challenge in advancing treatment strategies to repair the damaged spinal cord effectively.

Ultrasound offers promise as a potential treatment modality for SCI with the added benefit of being non-invasive. It has the ability to penetrate deep structures and has already shown therapeutic potential in treating central nervous system (CNS) conditions such as Parkinson’s disease (Martínez-Fernández et al., 2020; Pineda-Pardo et al., 2022) and epilepsy (Lin et al., 2020; Lescrauwaet et al., 2022). While extensive research has been undertaken and numerous devices have been developed for the brain (Perolina et al., 2024), the use of ultrasound for SCI still requires optimization. Key areas that need to be addressed include developing specialized devices that take into account the anatomical complexity of the spinal cord.

In this review, we focus on SCI and examine the emerging role of ultrasound in its treatment. We begin by outlining key ultrasound parameters commonly reported in the literature and detail their importance in achieving specific biological phenomena. This is followed by a discussion of different phases of SCI and how ultrasound has been explored in targeting associated pathological processes in each instance. Finally, we highlight critical considerations for translating these findings into clinical practice.

## Search Strategy

This literature review encompasses articles published up to 30^th^ of April 2025, which were identified and selected through screening of the MEDLINE and Google Scholar databases. Keywords such as “spinal cord injury,” “traumatic brain injury,” “neuronal regeneration,” “ultrasound,” “therapeutic ultrasound,” “focused ultrasound,” “drug delivery,” “drug delivery systems,” “neuromodulation,” and “mechanotransduction” were used. Articles in which ultrasound was used as an imaging modality to aid in SCI treatment, such as studies involving ultrasound-guided intrathecal injections of pharmacological agents, were excluded. No restrictions were placed on the types of studies included.

## Principles of Ultrasound Therapy and Key Parameters

Ultrasound refers to sound waves with frequencies above 20 kHz. These high-frequency sound waves can penetrate tissues and interact with cellular structures, resulting in a range of therapeutic effects.

### Ultrasound parameters

At its most basic level, ultrasound exposure is reported in the literature using a set of key parameters defining the ultrasound wave’s characteristics and its delivery to biological tissues (Quarato et al., 2023). These parameters can influence physiological outcomes in the CNS, including neuronal stimulation, neuronal regeneration, or ablation of unwanted tissue.

#### Fundamental frequency

The fundamental frequency of ultrasound refers to the number of oscillations per second of the ultrasound wave, typically measured in kHz or MHz. The frequency determines the ultrasound wavelength and thus its ability to penetrate tissues. Higher frequencies correspond to shorter wavelengths and provide a more confined focal zone, enabling more localized therapeutic effects. However, higher frequencies also lead to greater acoustic attenuation, meaning the waves are more likely to be absorbed or scattered by tissue (O’Brien, 2007). This is relevant for applications such as transcranial focused ultrasound, where skull thickness can severely limit the effective range of higher frequencies (Keihani et al., 2024). Similar challenges exist for SCI applications, as attenuation through the vertebrae and surrounding soft tissues can restrict frequency selection, requiring a careful balance between targeting precision and sufficient penetration to reach spinal cord structures non-invasively.

#### Intensity

Intensity refers to the amount of power delivered over a specific area of tissue, typically expressed in watts per square centimeter (W/cm²) or milliwatts per square centimeter (mW/cm²). It quantifies the power density of the ultrasound wave at a particular point in the tissue and is critical in determining the biological effects of ultrasound exposure.

Intensity can influence the degree of tissue response, with higher intensities potentially causing more significant effects such as thermal damage or tissue ablation (Izadifar et al., 2020). In comparison, lower intensities may lead to more subtle effects such as cellular membrane deformation or neuromodulation (Legon and Strohman, 2024). As a result, controlling the intensity is crucial for tailoring ultrasound to achieve the desired therapeutic or experimental outcomes.

#### Pressure

In therapeutic ultrasound, pressure refers to the physical force the ultrasound waves exert on tissue. Ultrasound waves consist of alternating compressions and rarefactions (expansions), which create pressure fluctuations in the tissue through which the waves travel. These pressure fluctuations are responsible for generating both mechanical and thermal effects in the tissue.

Pressure is generally characterized by the peak positive pressure and peak negative pressure (PNP), which represent the maximum amplitude of the compressive and rarefactive phases of the ultrasound wave, respectively. For therapeutic ultrasound, PNP is often a key parameter, particularly in applications involving mechanical effects such as cavitation, tissue disruption, and cellular membrane deformation (Perolina et al., 2024).

#### Mechanical index

The mechanical index (MI) is a unitless parameter used to quantify the potential for mechanical effects resulting from ultrasound exposure. MI relates PNP with frequency as shown in Equation 1, with PNP expressed typically in MPa and frequency in MHz (Nadrljanski and Murphy, 2010).

Equation 1 - Mechanical index


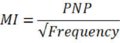


The MI serves as an indicator of the likelihood of cavitation—the formation of gas bubbles in tissue that can expand and collapse rapidly, generating localized high-energy events. These events can have significant mechanical effects on tissue, from transient reversible changes to cell membranes as seen with stable cavitation to damage to cellular structures or tissue necrosis as seen with inertial cavitation (Peruzzi et al., 2018).

A higher MI indicates a greater risk for cavitation and undesirable biomechanical effects. As such, it is a critical parameter when designing therapeutic ultrasound protocols, especially in settings where the potential for cavitation or tissue disruption must be controlled.

#### Pulse width, pulse repetition frequency, and pulse repetition period

**Figure 1:**
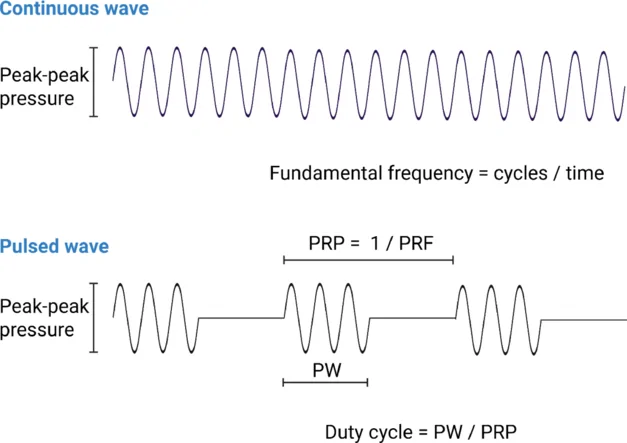
Visual representation of ultrasound parameters reported in literature. Created with BioRender.com. PRF: Pulse repetition frequency; PRP: pulsed repetition period; PW: pulse width.

Ultrasound waves can be delivered continuously or as pulsed waves. Pulse width (PW) and pulse repetition frequency (PRF) or pulse repetition period are key temporal parameters that are reported to describe the experimental ultrasound dose in literature (**Figure 1**). PW refers to the duration of an individual ultrasound pulse, typically measured in milliseconds. The duration of the pulse directly influences the amount of energy delivered to the tissue in each cycle. Shorter pulses (low PW) tend to result in more mechanical effects, such as cellular membrane deformation or vibration, which are often leveraged for neuromodulation or soft tissue treatments. Conversely, longer pulses (high PW) tend to generate more thermal effects due to sustained energy deposition, which can be used for tissue ablation or tumor treatment (Quarato et al., 2023).

PRF is the rate at which pulses are delivered during a sonication cycle, measured in Hz. PRF is the inverse of the pulse repetition period, which dictates how frequently ultrasound pulses are applied in a given timeframe.

#### Duty cycle

The duty cycle is a critical parameter in therapeutic ultrasound that refers to the proportion of time during a treatment pulse cycle that ultrasound energy is emitted. It is typically expressed as a percentage and is calculated as the ratio of the “on” time (when ultrasound energy is being emitted) to the total time of the pulse cycle (which includes both “on” and “off” periods). For example, a duty cycle of 50% means the ultrasound is on for half of the cycle and off for the other half.

#### Sonication duration and sonication intervals

Sonication duration refers to the total amount of time during which ultrasound energy is continuously delivered to the tissue in a single treatment session. It is typically measured in minutes and can vary depending on the therapeutic goal and the condition being treated. The duration of sonication directly influences the amount of energy that accumulates in the tissues. Longer sonication durations can be necessary to achieve significant thermal effects such as deep tissue heating. Shorter sonication durations are often used in cases where mechanical effects such as cavitation or microstreaming are the primary therapeutic focus, or when treating acute injuries where heat buildup could exacerbate inflammation.

Sonication intervals refer to the periods of rest or pause between each application of ultrasound energy during a treatment session. These intervals are important because they allow the tissue to recover between sonication periods, providing time for tissue healing or relaxation before additional energy is applied. The length of sonication intervals depends on the treatment parameters and the biological effects desired.

#### Initiation time in relation to injury and physiological healing mechanisms

Initiation time refers to when therapeutic ultrasound treatment begins after an injury, and its timing can significantly impact the body’s healing mechanisms. In the acute injury phase, early initiation of ultrasound can aid in the control of inflammation. In contrast, when ultrasound is applied in the chronic phase, after the inflammatory response has subsided, it can aid in tissue remodeling and reduce residual swelling. Understandably, the optimal parameters will vary depending on the desired bioeffect.

### Classification of therapeutic ultrasound modalities and regimes

Therapeutic ultrasound can be categorized by its intensity, focus, and delivery method. High-intensity ultrasound, such as high-intensity focused ultrasound (HIFU), is frequently utilized for targeted tissue ablation due to its capacity to produce pronounced thermal or mechanical effects at specific sites (Dubinsky et al., 2008). HIFU raises the temperature of the target tissue above 55 °C for at least 1 second, causing coagulative necrosis and immediate cell death (ter Haar and Coussios, 2007). This precise targeting creates a lesion with a characteristic histological appearance where the damaged area is surrounded by normal tissue. HIFU can also disrupt blood vessels, leading to hemorrhage (Rivens et al., 2024). The goal is to deliver rapid energy to the tissue, ensuring effective heating without significant cooling from blood flow or heat diffusion. In the CNS, HIFU has been used for targeting brain tissue in managing certain neurological conditions such as essential tremor (Rohani and Fasano, 2017) and Parkinson’s disease (Moosa et al., 2019).

Interestingly, in the context of SCI research, HIFU has been used not as a treatment, but rather as a method to induce SCI in small animal models, enabling the creation of reproducible and localized lesions for experimental studies (Oakden et al., 2014). Its destructive nature limits the therapeutic use of HIFU in SCI, where tissue preservation and tissue regeneration are key goals.

In contrast, low-intensity ultrasound, such as in low-intensity pulsed ultrasound (LIPUS) applications, is generally not considered capable of producing substantial thermal effects (Jiang et al., 2018). Furthermore, due to the relatively low acoustic output of LIPUS, cavitation effects are unlikely to occur unless exogenous agents such as microbubbles (gas-filled bubbles < 10 μm diameter) are introduced (Bader et al., 2025). Additionally, in therapeutic applications, pulsed ultrasound is often preferred for its ability to reduce thermal buildup. Unlike HIFU, low-intensity ultrasound offers the ability to modulate biological responses without causing overt damage, aligning more closely with the regenerative needs of the injured spinal cord.

### Basis of ultrasound bioeffects

The safety and efficacy of ultrasound therapies for SCI require careful consideration of the thermal and non-thermal (mechanical) effects. These effects can influence inflammation, neuronal survival, axonal regeneration, and vascular dynamics within the injured spinal cord. These bioeffects result from the interaction of propagating ultrasound waves with biological tissues, where energy is absorbed and converted into heat or transmitted as mechanical forces that affect cellular function and microenvironmental conditions. Understanding these mechanisms is crucial for developing ultrasound-based interventions aimed at neuroprotection, modulation of the injury, and enhancement of functional recovery following SCI.

#### Ultrasound attenuation and resulting thermal effects

Ultrasound waves travel through tissues via compression and rarefaction, creating pressure variations (Huang, 2020). As ultrasound waves propagate through tissue, several phenomena may contribute to the thermal effects. Attenuation refers to the gradual reduction in wave intensity due to reflection, absorption, and scattering. The extent of attenuation depends mainly on the physical properties of the tissue. Tissues with higher density, including vertebral bone, tend to attenuate ultrasound waves more efficiently. Conversely, tissues and media such as the cerebrospinal fluid, blood, and spinal cord parenchyma, which are rich in water, have lower attenuation and they propagate the ultrasound waves more effectively (Quarato et al., 2023). This principle is particularly important in spinal cord applications, where ultrasound must traverse bony structures, soft tissue, and fluid layers to reach the spinal cord, potentially limiting the ultrasound intensity delivered to the target or requiring full optimization of exposure parameters. Attenuation is typically quantified by the attenuation coefficient (Panetta and Demi, 2014) (α, Equation 2), which describes how much of the initial ultrasound intensity (I) is lost per unit distance travelled (*x*).

Equation 2 - Exponential attenuation of initial ultrasound intensity

*I*_*x*_ *= I*_*initial*_ × *e*^*–ax*^

The attenuation of ultrasound waves can be further described by outlining the phenomena that can occur as waves traverse through various tissue or media boundaries. The propagation of ultrasound waves through a specific tissue type or medium is influenced by the density of the tissue or medium (ρ) and the speed at which sound travels through the tissue or medium (c). Together, these characteristics are related and described as the acoustic impedance (Z, Equation 3).

Equation 3 - Acoustic impedance of a medium

*Z* = *ρc*

When ultrasound waves encounter boundaries between tissues of differing acoustic impedance, such as fat and muscle, part of the wave is reflected while the remainder continues to propagate. **Table 1** outlines the acoustic impedances of various tissues and media commonly used in disease modelling (i.e., cell or tissue culture vessels and tissue-mimicking phantoms).

**Table 1:** Acoustic impedance of cell culture ware, materials used for tissue-mimicking phantoms, and biological tissue

Material	Acoustic impedance ranges (kg/m^2^s)
Polystyrene	2.468 × 10^6^
Polypropylene	2.002 × 10^6^
Agarose	1.47–1.56 × 10^6^
Gelatin	1.53–1.59 × 10^6^
Polydimethylsiloxane	1.09–1.11 × 10^6^
Bone	7 × 10^6^
Skin	1.99 × 10^6^
Fat	1.38 × 10^6^
Brain	1.55–1.66 × 10^6^
Water	1.56 × 10^6^
Air	418

Data are sourced from Lochab and Singh (2004), Bruus (2012), Chen et al. (2022), and Hossein and Angeli (2023).

The pressure transmission (T_P_) and reflection (R_P_) coefficients are crucial for understanding the behavior of ultrasound waves at interfaces between different tissue types. T_P_ (Equation 4) is defined as the fraction of the energy of the wave that passes from one medium to the next, while R_P_ (Equation 5) is defined by the fraction of the energy from the ultrasonic wave that is reflected at the media boundary (Huang, 2020).

Equation 4 - Pressure transmission coefficient calculated using the impedance of two distinct media, *Z*_1_ and *Z*_2_


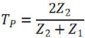


Equation 5 - Pressure reflection coefficient calculated using the impedance of two distinct media, *Z*_1_ and *Z*_2_


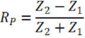


In addition to reflection, the tissue absorbs some of the ultrasound energy, converting mechanical energy into heat (Panetta and Demi, 2014). The extent of thermal effects is influenced by factors such as the intensity of the ultrasound wave, the duration of exposure, and the absorption capacity of the tissue. Like muscle, tissues with higher water content absorb energy more efficiently and heat up faster than tissues with lower water content, such as fat. While controlled heating can enhance blood flow, promote tissue elasticity, and accelerate cellular repair, excessive heating can cause tissue damage, making precise control of intensity and exposure duration essential. This absorption, which is responsible for the thermal effects of ultrasound, plays a key role in therapeutic applications, such as treating muscle spasms and tissue ablation in the case of HIFU. Scattering further diminishes the intensity of the waves as they pass through the tissue, with denser and less homogeneous tissues such as bone or collagen-rich tendons causing more significant attenuation.

Taken together, the phenomena of reflection, absorption, and scattering collectively explain how ultrasound waves interact with tissues. These principles are critical for understanding both the limitations and potential of ultrasound as a therapeutic tool, particularly in complex tissues such as the spinal cord. Attenuation reduces the energy of ultrasound waves as they propagate through tissues, impacting not only the depth of penetration but also the intensity at the target site, thereby influencing the distribution of thermal effects.

#### Mechanical effects of ultrasound

The mechanical effects of ultrasound occur when the momentum from the ultrasound wave is propagated to tissues or other media, leading to phenomena such as acoustic radiation force, fluid streaming, and cavitation. Acoustic radiation force results in the physical displacement of tissues (Kim et al., 2023), while fluid streaming refers to the movement of biofluids caused by the ultrasound wave (Peng et al., 2021). Both effects are influenced by factors such as frequency, *in situ* intensity, tissue attenuation, and viscosity of the medium the wave is traversing through.

Cavitation, another key mechanical phenomenon, arises from the interaction of ultrasound with gaseous nuclei (Lee et al., 2020), such as endogenous or exogenous microbubbles. Cavitation can be classified into two types: inertial and stable. Inertial cavitation occurs when microbubbles rapidly collapse after expanding significantly during the negative pressure phase of the ultrasound wave, producing localized high-energy effects that can damage surrounding structures (Zhao et al., 2013). In contrast, stable cavitation involves gentle, sustained oscillations of microbubbles, which are less likely to cause tissue damage (Caskey et al., 2007). In the context of SCI, stable cavitation may be harnessed to modulate the injury microenvironment by enhancing drug delivery, altering blood–spinal cord barrier (BSCB) permeability, or stimulating mechanotransduction pathways without inducing further damage. The occurrence of cavitation is influenced by factors such as PNP, ultrasound frequency, PRF, and the availability of cavitation nuclei (Cheng et al., 2019). These mechanical effects, alongside thermal effects, are essential for achieving therapeutic outcomes and can occur simultaneously under certain ultrasound exposure conditions.

#### Potential mechanisms behind the effects of ultrasound on neuronal cells

A potential mechanism behind the effects of ultrasound on neuronal cells involves the interaction of ultrasound with the cell membrane. Ultrasound waves can induce mechanical strain on the membrane, causing cyclic contraction and expansion. This strain modulates the gating and conformation of ion channels within the membrane, altering ionic flux and thereby influencing neuronal activity (Prieto and Maduke, 2024).

The bilayer sonophore model, which integrates bubble dynamics with cell biomechanics, further proposes that ultrasound-induced membrane motion can initiate signaling cascades leading to downstream biological effects (Krasovitski et al., 2011). Furthermore, low-frequency ultrasound application has been shown to lead to the nucleation of stable gas bubbles within the lipid bilayer (Wrenn et al., 2013). These bubbles form when gas molecules partition from the surrounding aqueous medium into the bilayer core, disrupting the lipid packing and creating microscale bubbles. The presence of these bubbles can further contribute to membrane disruption and permeability. The model predicting bubble nucleation shows that the probability of stable bubble formation is highly sensitive to bilayer thickness, and the presence of these bubbles may be responsible for the observed effects of low-frequency ultrasound on the membrane. These mechanisms offer promising therapeutic applications such as CNS modulation and BSCB permeabilization.

## Place of Ultrasound Therapy in the Different Phases of Spinal Cord Injury

The pathological events in SCI are typically divided into primary injury and secondary injury, with the latter progressing through acute, subacute, and chronic phases. Each stage involves distinct mechanisms that contribute to the progression of damage. Preclinical studies suggest that ultrasound may influence inflammation, neurotrophic factor expression, angiogenesis, glial scar formation, and even neuronal activity. Compared to other treatment modalities, ultrasound offers the advantages of being non-invasive and compatible with real-time neuroimaging techniques such as magnetic resonance imaging (Liao et al., 2021b). Additionally, it can be readily combined with other therapies, including electrical stimulation and co-delivered pharmaceuticals (Xu et al., 2024; Wang et al., 2025a). Finally, ultrasound is desirable as the technique can achieve millimeter-level targeting accuracy and precision (Liao et al., 2021b). Despite encouraging early results, the direct evidence of ultrasound’s therapeutic efficacy in SCI remains limited. In this section, we review pathophysiological features of SCI, and for each stage, highlight the supporting pre-clinical data and proposed mechanisms through which ultrasound might contribute to functional recovery or repair.

### Primary injury

Primary injury refers to the initial trauma that directly disrupts the spinal cord’s structural integrity (Alizadeh et al., 2019). This can include mechanical damage to axons, glial cells, the BSCB, and spinal vasculature. Examples include compression, laceration, or contusion of the spinal cord due to traumatic events such as motor vehicle accidents, falls, or sports injuries. The extent of damage depends on the trauma’s force and duration, with axonal severance and vascular disruption being key features. This initial insult sets the stage for secondary injury mechanisms that amplify the damage (Anjum et al., 2020). Ultrasound, if applied in the primary injury phase is postulated to exert neuroprotective effects, potentially reducing the severity of secondary injury (Yulug et al., 2017).

### Secondary injury: Acute, subacute, and chronic phases

Secondary injury begins immediately after the primary trauma and evolves over hours, days, and weeks. It is characterized by a cascade of cellular and molecular processes that exacerbate the damage to the spinal cord.

#### Inflammation in the acute phase

During the acute phase, the rupture of blood vessels leads to severe hemorrhage, edema, and disruption of the BSCB. This allows an influx of inflammatory cells, such as neutrophils and macrophages, into the lesion site (Sterner and Sterner, 2022). Macrophages have been classified into M1 and M2 groups. M1 macrophages produce pro-inflammatory cytokines, reactive oxygen species, and nitric oxide, driving a localized inflammatory response. Conversely, M2 macrophages are characterized by their anti-inflammatory functions and have a limited capacity to generate pro-inflammatory mediators such as inducible nitric oxide synthase and tumor necrosis factor-α. In SCI, the macrophage population is predominantly composed of pro-inflammatory M1 macrophages, with relatively few anti-inflammatory M2 macrophages present (Wang et al., 2015).

**Figure 2:**
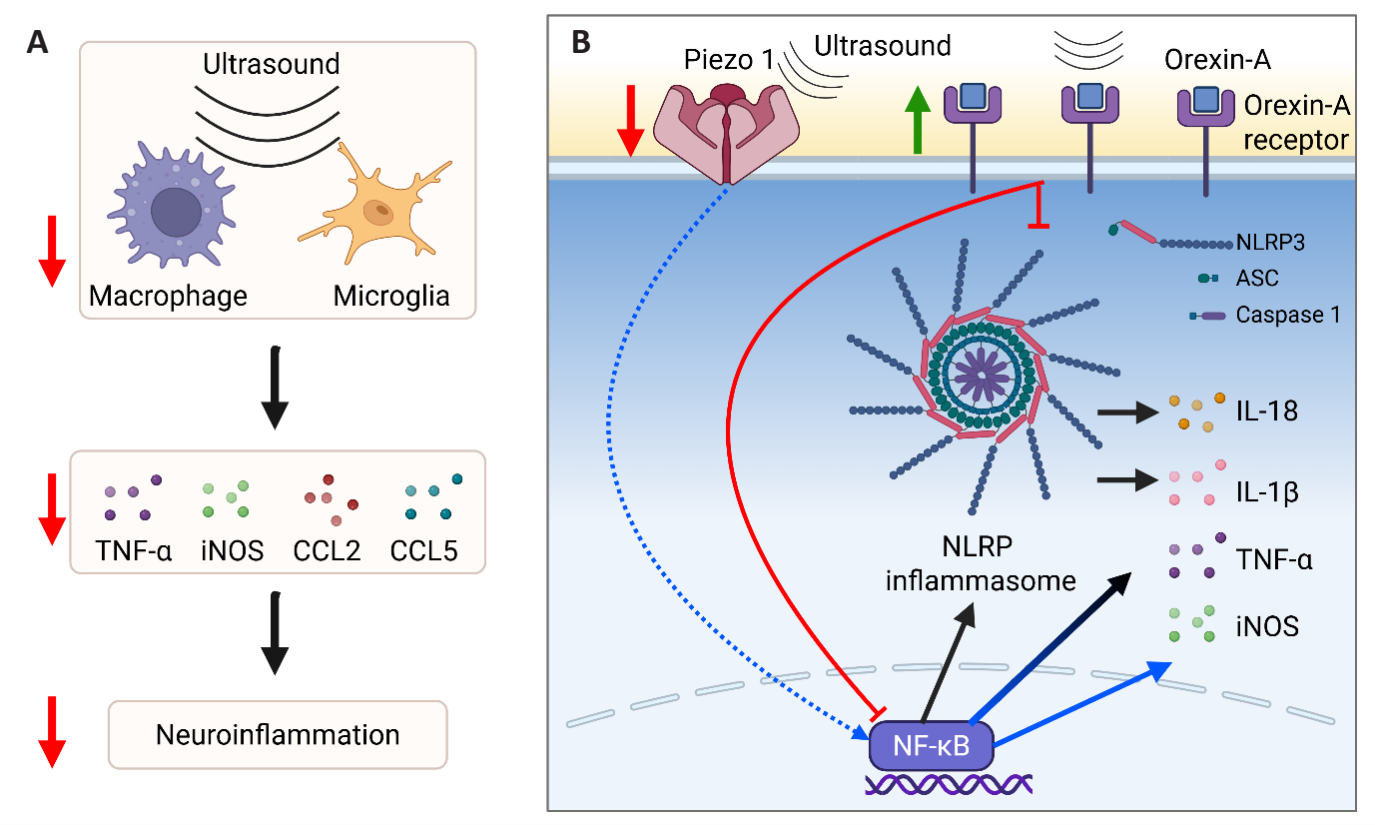
Ultrasound-mediated modulation of neuroinflammation. (A) Ultrasound exposure reduces activation of macrophages and microglia, leading to decreased expression of pro-inflammatory markers including TNF-α, iNOS, CCL2, and CCL5. This results in an overall reduction in neuroinflammation. (B) At the cellular level, ultrasound inhibits the mechanosensitive Piezo1 channel (red down arrow), which in turn reduces activation of the NF-κB pathway and downstream pro-inflammatory mediators. Ultrasound also enhances Orexin-A signaling via its receptor (green up arrow), which contributes to suppression of the NLRP3 inflammasome. Together, these pathways act on NF-κB to attenuate neuroinflammatory responses. Components of the NLRP3 inflammasome include NLRP3, ASC, and caspase-1, which drive the maturation of IL-1β and IL-18. This schematic illustrates the dual regulatory effects of ultrasound on inflammation through mechanical and neuropeptidergic mechanisms. Created with BioRender.com. ASC: Apoptosis-associated speck-like protein containing a CARD; CCL: C-C motif chemokine ligand; IL: interleukin; iNOS: inducible nitric oxide synthase; NF-κB: nuclear factor kappa B; NLRP: nucleotide-binding oligomerization domain, Leucine rich Repeat and Pyrin domain containing Proteins; TNF-α: tumor necrosis factor-α.

Pulsed ultrasound therapy has been shown to reduce the expression of ED-1, a marker for macrophages and microglia, in a rat model of SCI. This reduction in ED-1 expression was associated with a decrease in pro-inflammatory markers, including inducible nitric oxide synthase and tumor necrosis factor-α (Hong et al., 2022; **Figure 2**). These findings suggest that ultrasound may exert its effects through mechanisms involving macrophage activation, potentially influencing the polarization of macrophages from the pro-inflammatory M1 phenotype to the anti-inflammatory M2 phenotype.

This reduction in pro-inflammatory mediators was consistent in an SCI rat study utilizing pulsed ultrasound therapy at higher acoustic pressure, duty cycle and prolonged pulse repetition period (Zhang et al., 2023). This study suggested that the upstream mechanism may involve the downregulation of the mechanosensitive protein Piezo1, although the specific downstream mechanisms, particularly those involving the inhibition of nuclear factor-κB signaling (**Figure 2**), were not fully elucidated. An alternative mechanism was proposed in a traumatic brain injury study, which suggested that ultrasound can reduce inflammation by upregulating orexin-A and orexin-A receptor expression. Orexin-A has previously been shown to prevent neuronal death and support regeneration in other CNS conditions, such as intracerebral hemorrhage and Alzheimer’s disease. By increasing orexin-A expression, ultrasound significantly inhibited the activation of nuclear factor-κB and the NLRP3 inflammasome following injury, leading to a reduction in the release of pro-inflammatory factors tumor necrosis factor-α, interleukin-1β, and interleukin-18 (**Figure 2**; Huang et al., 2022).

#### Acute axonal degeneration

Ischemia, inflammation, and oxidative stress lead to neuronal necrosis and acute axonal degeneration, a key pathological feature of SCI (Alizadeh et al., 2019). Acute axonal degeneration is driven by a surge in intracellular calcium, triggering two stages: an early phase within 15 minutes and Wallerian degeneration over 24–48 hours. Wallerian degeneration involves retraction bulb formation, inhibiting axonal regeneration, while axonal dieback further contributes to neuronal dysfunction. Intrinsically, mature neurons have limited growth capacity as they downregulate growth-associated genes such as growth associated protein 43 and small proline-rich protein 1A. Furthermore, mature neurons have insufficient neurotrophic support with growth factors such as brain-derived neurotrophic factor (BDNF), nerve growth factor, and neurotrophin-3.

**Figure 3:**
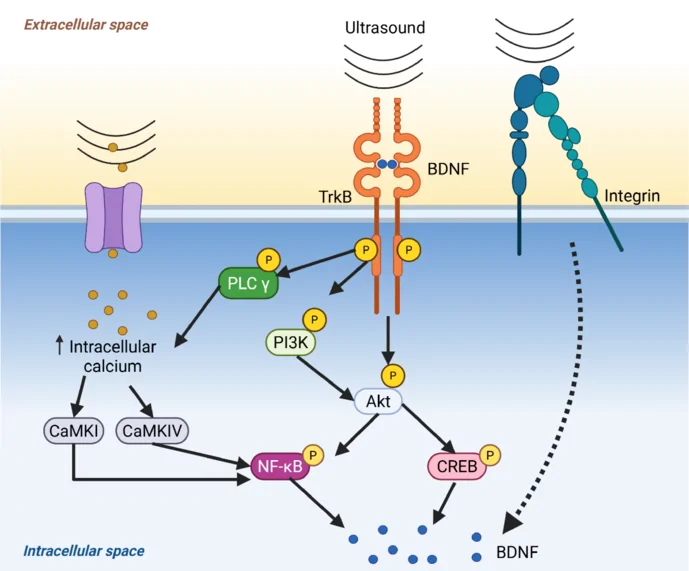
Ultrasound stimulation promotes BDNF signaling through integrin- and TrkB-mediated intracellular pathways. Ultrasound activates cell surface receptors, including integrins and TrkB, leading to phosphorylation-dependent signaling cascades. TrkB activation by BDNF (brain-derived neurotrophic factor) stimulates the PI3K-Akt and PLCγ pathways. PLCγ activation increases intracellular calcium, which activates CaMKI and CaMKIV, further promoting NF-κB signaling. Simultaneously, the PI3K-Akt pathway activates CREB, enhancing BDNF expression in a positive feedback loop. Integrins also contribute to TrkB activation and BDNF signaling amplification. Created with BioRender.com. BDNF: Brain-derived neurotrophic factor; CaMK: calcium/calmodulin-dependent protein kinase; CREB: cAMP response element-binding protein; NF-κB: nuclear factor kappa B; PI3K: phosphoinositide 3-kinase; PLCγ: phospholipase C-gamma; TrkB: tyrosine receptor kinase B.

Ultrasound has a place in therapy here as it has been postulated to upregulate endogenous growth factors in the CNS (**Figure 3**). Ultrasound stimulation shows promise in promoting axonal regrowth by enhancing cellular signaling through interactions with mechanosensitive proteins. In a mouse model of traumatic brain injury, ultrasound stimulation increased protein levels of BDNF and vascular endothelial growth factor (VEGF) (Su et al., 2017). This enhancement led to increased phosphorylation of tyrosine receptor kinase B (Trk B), Akt, and cAMP response element-binding protein, ultimately reducing apoptosis. While the specific mechanisms by which ultrasound increases BDNF expression in this study were not fully elucidated, existing evidence suggests that ultrasound interacts with integrins, mechanosensitive proteins on cell surfaces, to promote neurotrophic expression in glial cells such as astrocytes. A different *in vitro* study on rat astrocytes showed that the ultrasound-induced expression of BDNF, glial-derived neurotrophic factor, and VEGF was suppressed when an integrin inhibitor (disintegrin RGD peptide) was introduced (Yang et al., 2015). This suggests that ultrasound may activate neurotrophic factor expression through integrin receptor signaling.

In addition to ultrasound’s interaction with integrins, a separate study reported ultrasound interactions with other ion channels that led to a downstream increase in BDNF expression, alongside TrkB and Akt signaling (Liu et al., 2017). Immunoblotting data from the study revealed that ultrasound stimulation significantly increased the protein expression of phosphorylated TrkB, phosphorylated Akt, and PLC-γ in astrocytes. Additionally, ultrasound stimulation transiently elevated intracellular calcium levels, suggesting that the TrkB/PI3K/Akt and TrkB/PLC-γ/Ca^2+^ signaling pathways may be involved in regulating astrocyte function in response to ultrasound.

#### Demyelination and loss of oligodendrocytes in acute and subacute phases

Demyelination is a pathological feature of SCI, and it causes disruption in nerve signal conduction and weakens axonal integrity (Almad et al., 2011). Oligodendrocytes, a type of glial cell responsible for producing myelin, are highly vulnerable to glutamate excitotoxicity. Excessive receptor activation and calcium influx lead to widespread oligodendrocyte loss and further exacerbate neuroinflammation. In conditions where demyelination is a pathological feature, such as multiple sclerosis, ultrasound has been shown to accelerate remyelination by promoting the maturation of oligodendrocyte progenitor cells, thereby enhancing their density and BDNF production (Yang et al., 2022).

#### Vascular disruption in subacute phase of spinal cord injury

The subacute phase is marked by ongoing ischemia caused by persistent edema and compromised vasculature (Anjum et al., 2020). This leads to oxygen deprivation, metabolic disturbances such as lactic acid accumulation, and ultimately necrosis or apoptosis of neurons and oligodendrocytes, contributing to the severity of injury. While endogenous angiogenesis is triggered, the newly formed vessels are often structurally abnormal and functionally inadequate, exacerbating inflammation and hindering recovery. Low-intensity ultrasound has been shown to upregulate the expression of VEGF, endothelial nitric oxide synthase, and basic fibroblast growth factor in ischemic tissues. These factors play pivotal roles in promoting endothelial cell differentiation and vessel maturation, collectively enhancing angiogenesis. In the context of nerve injury, ultrasound has again been shown to upregulate the expression of VEGF as well as its receptors, leading to the formation of new vessels post-injury in peripheral nerves (Hizay et al., 2022). In a traumatic brain injury model, focused ultrasound promoted VEGF expression, encouraged cerebral vascular remodeling in the brain injury area, and reduced secondary brain injury caused by hypoxia and ischemia. This ultimately led to improved neurological outcomes following injury (Yi et al., 2024).

#### Formation of the astro-glial scar

A complex interplay among various glial cells leads to the formation of an astro-glial scar, which serves to contain damage but also inhibits neural regeneration. Initially, astrocytes become reactive, proliferate, and secrete molecules such as glial fibrillary acidic protein and chondroitin sulphate proteoglycans, contributing to the scar’s structure and impeding axonal regrowth (Bradbury and Burnside, 2019). Microglia are activated, releasing cytokines that exacerbate inflammation but also aid in debris clearance (Xu et al., 2021). Oligodendrocytes may undergo apoptosis, resulting in demyelination, while oligodendrocyte precursor cells proliferate and migrate to the injury site; however, their differentiation is often hindered by the inhibitory environment of the scar (Li and Leung, 2015). Additionally, the injury often leads to hemorrhage and necrosis, resulting in the formation of fluid-filled cystic cavities within the lesion site. These cavities progressively expand and coalesce, further contributing to cell death and impeding axonal regeneration. This coordinated response forms a barrier that protects surrounding tissue but poses significant challenges for neural repair and functional recovery (Alizadeh et al., 2019; Anjum et al., 2020).

Ultrasound therapy has the potential to address the events that lead to the formation of the astro-glial scar through enhancing drug, cell, and gene delivery. Focused ultrasound, particularly when combined with microbubbles, can transiently increase the permeability of the BSCB. This increased permeability facilitates the delivery of therapeutic agents directly to the injury site, potentially improving the effectiveness of drug and gene therapies aimed at neural repair (Perolina et al., 2024).

Preclinical evidence suggests that ultrasound can aid in the effective transplantation of stem cells for the treatment of SCI. Ultrasound stimulation of bone marrow mesenchymal stem cells prior to transplantation enhanced their viability and neurotrophic factor expression levels *in vitro*; transplantation of these pre-stimulated bone marrow mesenchymal stem cells resulted in reduced cavity formation and improved locomotor function in rats (Ning et al., 2019). Neural stem cells encapsulated in a piezoelectric hydrogel, along with Schwann cells, were co-delivered and exposed to ultrasound stimulation. The ultrasound stimulation promoted neural stem cell differentiation into neurons and supported remyelination through activation of the piezoelectric properties of the hydrogel (Wang et al., 2025a). Future studies could include specifically using ultrasound to promote the differentiation of oligodendrocyte precursor cells in a similar manner for SCI treatment. This has been conducted previously in a mouse model of ischemic stroke (Wang et al., 2025b).

As discussed earlier, ultrasound stimulation has been observed to promote the resolution of inflammation and provide neuroprotection in SCI models (Hong et al., 2022; Su et al., 2023). By modulating the inflammatory response, ultrasound may reduce the inhibitory effects of the astro-glial scar, creating a more conducive environment for axonal regeneration and functional recovery.

#### Chronic phase, maturation of the glial scar and secondary complications

In the chronic phase of SCI, pathological changes become more stable but also more inhibitory to recovery. In these cases, neurostimulation methods have been used to restore function and to treat pain (James et al., 2018). Ultrasound, particularly at low intensities, has shown benefit in SCI as a neurostimulation modality, either by direct interactions with mechanosensitive proteins in the spinal cord, or paired with piezoelectric stimulators, which convert acoustic mechanical energy to electrical signals.

Previous work applying low-intensity ultrasound to the motor cortex activated neurons and evoked motor behavior as confirmed by electromyography (Liu et al., 2023). Similar findings were observed when ultrasound was applied to stimulate L4–L5 segments of the uninjured rat spinal cord, with the induction of soleus muscle contraction as confirmed by electromyography (Liao et al., 2021b). This non-invasive modulation of motor function was also replicated in a motor deficit mouse model, where sonication of the lumbar-3 segment had a facilitatory effect on electromyography activity (Kim et al., 2022). These studies indicate that ultrasound can be used as a non-invasive neurostimulation method to restore motor function following SCI. Mechanistic studies found that ultrasound works non-invasively to modulate neuronal activity by activating voltage-gated sodium and calcium channels, as well as mechanosensitive ion channels such as Piezo1/2 in the brain (Song et al., 2023). However, further studies are required to fully elucidate, which mechanisms govern ultrasound neuromodulation in SCI contexts.

Aside from directly stimulating neurons, ultrasound has also been shown to work in conjunction with other neuromodulation methods. For instance, ultrasound can be paired with piezoelectric stimulators to restore locomotion. Other neurostimulation methods require an external power source to work effectively, with most implantable stimulators requiring batteries, thus increasing the size of the implant. Ultrasound can mitigate this by interacting with piezoelectric materials that convert the received mechanical signal to electrical signals. This method has been tested in a rat model of SCI (Alam et al., 2019; Li et al., 2020), where the ultrasound-powered piezostimulator electrodes implanted on lumbar 2 and sacral 1 segments of the cord induced motor-evoked potentials, restoring locomotion.

## Use of Ultrasound for the Treatment of the Secondary Complications of Spinal Cord Injury

SCI leads to a range of secondary complications that significantly impact the patient’s quality of life, including neuropathic pain, spasticity, bladder and bowel dysfunction, and impaired wound healing. Preclinical data suggest that ultrasound-based therapies offer a potential treatment option for managing these complications; however, additional research is needed before any clinical benefits are realized.

### Neuropathic pain

Neuropathic pain is a common consequence of SCI and is often resistant to conventional analgesics (Hagen and Rekand, 2015). The molecular mechanism behind SCI-induced neuropathic pain remains to be fully understood; however, a preclinical study suggests that the dysregulation of chloride homeostasis through the downregulation of ionic co-transporters such as the potassium chloride co-transporter 2 (KCC2) may be implicated (Talifu et al., 2022). One study found that low-intensity focused ultrasound (LIFU) increased KCC2 expression while inhibiting the calcium/calmodulin-dependent protein kinase IV-KCC2 pathway in the L4–L5 spinal cord, thereby restoring chloride homeostasis and reducing pain hypersensitivity in rodent models (Liao et al., 2021a).

The dysregulation of chloride homeostasis can further result in the transformation of GABAergic neurons from inhibitory to excitatory, leading to neuropathic pain (Talifu et al., 2022). LIFU has been previously found to modulate GABAergic and calcitonin gene-related peptide pathways in peripheral sensory neurons to provide time-dependent analgesia, offering potential for non-invasive pain management (Lin et al., 2025). While these effects are promising, the molecular mechanisms behind neuropathic pain need to be researched further (Shiao and Lee-Kubli, 2018); simultaneously, more studies are needed to clarify the role of ultrasound as a treatment option for neuropathic pain.

### Spasticity

Spasticity, characterized by involuntary muscle stiffness and spasms, is a major challenge for individuals with SCI. As with neuropathic pain, it arises from an imbalance in excitatory and inhibitory signaling within spinal and supraspinal motor pathways. A key contributor to this imbalance is the disruption of chloride homeostasis, particularly due to the downregulation of potassium-chloride co-transporter KCC2 in spinal motoneurons (Boulenguez et al., 2010). This impairs inhibitory GABAergic transmission, leading to motor neuron hyperexcitability and the development of spasticity (Bhagwani et al., 2022).

Preclinical research indicates that ultrasound can modulate spinal and supraspinal circuits involved in spasticity. A study in rats found that low-intensity ultrasound effectively activates spinal cord neurocircuits, reduces spasticity, and enhances the expression of KCC2 non-invasively (Liao et al., 2022). A separate study demonstrated that LIPUS applied to the spinal cord of rats significantly suppressed hindlimb motor evoked potential amplitudes during sonication, with levels gradually returning to baseline post-sonication. This suppression indicates that ultrasound can modulate motor signals within the spinal cord, suggesting its potential in treating movement disorders characterized by excessive neuronal excitation (Tsehay et al., 2023).

### Bladder dysfunction

Neurogenic bladder dysfunction is a significant complication of SCI, often presenting as neurogenic detrusor overactivity. This results in incontinence, urinary retention, and recurrent infections. The pathophysiology is multifactorial, involving disrupted neural communication between the brain, spinal cord, and bladder, with an imbalance between excitatory and inhibitory reflexes governing the micturition reflex. Following SCI, plastic changes in afferent and efferent pathways, as well as maladaptive spinal reflexes, contribute to the development of bladder overactivity and dysfunction (Perez et al., 2022).

Emerging evidence suggests that neuromodulation techniques, including low-intensity ultrasound, may offer a non-invasive therapeutic avenue to restore control over bladder function. Preclinical studies have shown that ultrasound stimulation of peripheral nerves involved in micturition control can modulate bladder reflexes. One study demonstrated that LIFU applied to the tibial nerve in rats significantly suppressed bladder overactivity (He et al., 2025). This effect is thought to occur through modulation of afferent sensory signaling and reflex arcs responsible for bladder activity. While these initial findings are promising, the precise mechanisms by which ultrasound affects bladder control, particularly in the context of SCI, remain to be fully investigated. Further studies are needed to define the optimal stimulation parameters, targets, and long-term outcomes of the ultrasound-based neuromodulation for neurogenic bladder dysfunction.

### Wound healing

Pressure ulcers and chronic wounds are significant and often persistent complications following SCI, largely due to impaired mobility, reduced sensation, and compromised vascular perfusion (Vecin and Gater, 2022). These wounds frequently develop at weight-bearing sites and are exacerbated by prolonged immobility, friction, shear forces, and autonomic dysfunction that impairs thermoregulation and tissue oxygenation. The healing of such wounds is often delayed in individuals with SCI due to chronic inflammation, poor angiogenesis and diminished cellular activity within the wound bed (Rappl, 2008).

Ultrasound has been widely studied for its role in promoting tissue healing and regeneration. Preclinical studies have shown that LIPUS enhances angiogenesis, reduces inflammation, and accelerates wound closure in animal models of skin injury (Yadollahpour et al., 2014). Several studies have demonstrated that LIPUS increases blood flow, stimulates fibroblast activity, and enhances collagen deposition, leading to faster wound healing (Maan et al., 2014; Iwanabe et al., 2016; Chen et al., 2019; Wang et al., 2025d).

## Considerations for Clinical Translation

Most available data supporting the beneficial effects of ultrasound in SCI therapy have been generated in preclinical models. To move this promising therapeutic approach closer to the clinic, several factors need to be optimized to ensure safety and therapeutic efficacy. This section will cover the following components: ultrasound parameters, transducer selection, coupling methods, positioning, and treatment planning, all of which require careful consideration of anatomical features.

### Safe and effective ultrasound parameters

Extensive animal studies have highlighted the importance of controlling ultrasound parameters to prevent tissue damage (Pasquinelli et al., 2019; Lee et al., 2021). Key parameters influencing safety include spatial-peak time-averaged intensity (I_SPTA_), exposure duration, duty cycle, PRF, and fundamental frequency (Blackmore et al., 2019). Higher intensities and prolonged exposure times are associated with increased risks of thermal and mechanical damage, including hemorrhage, necrosis, and inflammation.

A comprehensive safety review analyzed 16 preclinical studies using various animal models to determine the threshold at which ultrasound exposure may cause spinal cord damage (Xu et al., 2024). By plotting I_SPTA_ against total exposure time in log–log space, the study identified a power-law relationship representing the minimum reported damage thresholds. This relationship is described by Equation 6.

Equation 6 – Intensity and exposure time threshold for tissue damage in spinal cord ultrasound applications

*I*_*SPTA*_ = 37 × (*exposure time*) ^–0.77^

where I_SPTA_ is measured in W/cm² and exposure time is measured in seconds. This equation can be used to estimate the minimum I_SPTA_ that may cause damage for a given exposure time or can be rearranged to determine the minimum exposure duration that could result in damage at a specified intensity. For BSCB opening, the study found that the introduction of ultrasound contrast agents to facilitate the barrier disruption made it so that the relationship between intensity and time offers no utility in predicting damage as the damage in this instance is more likely due to cavitation rather than thermal effects.

Aside from acoustic intensity and exposure time, duty cycle and PRF must also be carefully optimized to balance therapeutic efficacy and safety. Higher duty cycles and lower PRFs can increase the risk of cumulative thermal effects. Furthermore, increasing these values could lead to different, but not necessarily preferred outcomes altogether. For example, a study on LIFU in rats with SCI found that a 5% duty cycle improved motor function and reduced tissue damage more effectively than a 40% duty cycle (Hong et al., 2022). Similarly, in a human study on spinal cord ultrasound stimulation, high-intensity stimulation (10 W/cm²) with a 30% duty cycle and a 1 kHz PRF significantly reduced motor-evoked potentials compared to sham stimulation; however, reducing the duty cycle to 10% did not diminish these outcomes (Hou and Lei, 2025). Additionally, PRF optimization is critical, as ultrasound has been shown to suppress spinal reflexes in a PRF-dependent manner: higher PRFs enhanced homosynaptic depression of the H-reflex, while lower PRFs suppressed spinal reflexes (Song et al., 2024).

Acoustic frequency also plays a role, with lower frequencies generally offering deeper penetration. In rodent models of spinal cord neuromodulation, ultrasound frequencies between 0.5 and 4 MHz have been previously used in clinical studies with different applications (**Table 2**). It is worth noting that human spinal cords and vertebrae are larger and more dense than commonly used animal models. As such, careful consideration of transducer design and anatomy is warranted.

**Table 2:** Ultrasound parameters used for preclinical studies on different spinal cord injury models and related applications

Animal model and application	Frequency	Pressure or intensity	Duty cycle	PRF	Exposure time/interval	Efficacy outcomes	Safety outcomes	Reference
Rat (thoracic SCI)	1 MHz	0.8 W/cm^2^	5%, 40%	1 kHz	5 min/d on d 2 to 4 post-injury (3 times total)	5% duty cycle protocol improved hindlimb function, reduced lesion cavity and observable histological damage and had anti-inflammatory effects. Lesser improvements in motor function in 40% duty cycle protocol.	No additional damage to the cord reported.	Hong et al., 2022
Rat (cervical SCI)	1 MHz	67.35 mW/cm^2^ I_SATA_ in water	20%	1 kHz	5 min	Low-intensity pulsed ultrasound improved forelimb strength, forelimb reaching and grasping function.	No adverse effects reported.	Ahmed et al., 2023
Rat (thoracic SCI, with transplantation of neural stem cells)	1 MHz	100 mW/cm^2^	60%	n.r.	Ultrasound applied 1 wk post-injury and transplantation	Low-intensity pulsed ultrasound improved the proliferation, differentiation and survival of neural stem cells transplanted at the SCI site. This in turn improved the functional outcomes of the SCI rats.	No adverse effects reported.	Liao et al., 2025
Rat (BSCB opening for drug delivery for SCI)	940 kHz	1–2 MPa	2.5%	1 Hz	3 min (3 times in total)	BSCB selectively opened in both injured and non-injured spinal cords to facilitate drug delivery as confirmed using magnetic resonance imaging and dye-based evaluations.	No adverse effects reported.	Cross et al., 2021
Rabbit (BSCB opening feasibility study, no SCI, laminectomy)	1.1 MHz	0.3–0.8 MPa nominal pressure with multiple segments sonicated	Continuous – n/a	Continuous – n/a	150 s	Effective localized opening of the BSCB confirmed using Evans Blue permeation. Effect more pronounced within gray matter and in rabbits ventilated with ambient air compared to those ventilated with isoflurane–oxygen.	43% of sonicated segments exhibited moderate to severe histologic lesions, including hemorrhage and structural disruption, particularly when surgical trauma was present or at higher ultrasound exposures.	Montero et al., 2019
Rat (BSCB opening feasibility study, no SCI)	250 kHz	0.47 MPa	40%	1 kHz	5 min	The combined use of low-intensity focused ultrasound and microbubbles successfully opened the BSCB.	Histology showed no neuronal damage or structural lesions, but occasional small hemorrhages at therapeutic ultrasound levels. Temperature increases were minimal (≤ 1.3°C), suggesting a low risk of thermal injury. At higher intensities, tissue damage, including hemorrhage, cavities, and vacuolization, was observed.	Bhimreddy et al., 2023
Rat (SCI neuromodulation)	4 MHz	0.65 MPa	50%	0.8 kHz	20 min/d for 4 wk, starting 1 wk post-SCI	Ultrasound stimulation promoted functional recovery after SCI. After 4 wk, ultrasound-treated rats had higher mechanical thresholds and improved BBB scores, though still below sham levels. Ultrasound also reduced spasticity. At the molecular level, ultrasound increased KCC2 expression in lumbar spinal neurons.	Safety outcomes not reported.	Liao et al., 2022
Rat (SCI, neuromodulation for spasticity)	1 MHz	0.68 MPa	20%	0.8 kHz	20 min/d for 4 wk, starting 1 wk post-SCI	Following 4 wk of ultrasound treatment, the spastic response improved with reduced muscle stiffness and tension of the lower limbs and tails.	No adverse effects reported.	Wang et al., 2023
Rat (feasibility study for using ultrasound neuromodulation for treating chronic spine-related conditions such as SCI)	4 MHz	0.5–3 MPa	20%	1 kHz	20 min	Stimulation of the L4–L5 spinal cord segments enhanced soleus muscle contraction.	At 3.0 MPa, ultrasound caused spinal cord damage, including reduced somatosensory evoked potentials, necrosis, structural damage, and neuronal loss in the dorsal horn. Intensities ≤ 1.5 MPa were safe and effectively activated spinal neurocircuits without causing damage.	Liao et al., 2021b
Rat (feasibility study for using ultrasound neuromodulation for movement disorders driven by excessive excitation of spinal neurons such as SCI)	1.1 MHz	0.83–3 MPa pressures tested	40%	2 kHz	1 min	Ultrasound stimulation reversibly suppressed spinal reflexes in a pressure- and PRF-dependent manner. Ultrasound also increased withdrawal reflex latency.	3 MPa could potentially produce lesions, and thus, lower pressures were used.	Song et al., 2024
Motor deficit mouse model for studying feasibility of using transpinal focused ultrasound to modulate descending motor pathways	3 MHz	T12: 2.2, 1.4, 0.8 MPa L3: 2.2 and 1.4 MPa	50%	1 kHz	n.r.	Focused ultrasound modulated motor pathways non-invasively, with effects depending on the spinal region and acoustic pressure. Thoracic sonication caused inhibition, while lumbar sonication facilitated motor activity. Effects were transient, returning to baseline within minutes. Ultrasound at T12 significantly reduced tremors.	Temperature elevation is below threshold for damage for neuronal cells. No hemorrhage or neural death in histological data.	Kim et al., 2022

BBB: Basso–Beattie–Bresnahan; BSCB: blood–spinal cord barrier; Hz: hertz; I_SATA_: spatial-average temporal-average intensity; KCC2: potassium–chloride cotransporter 2; kHz: kilohertz; L: lumbar (spinal segment); MPa: megapascal; mW/cm^2^: milliwatts per square centimeter; n.r.: not reported; SCI: spinal cord injury; T: thoracic (spinal segment); W/cm^2^: watts per square centimeter; wk: week.

The use of low-intensity ultrasound in SCI therapies is an emerging area of research, with preclinical studies showing positive outcomes in terms of motor function recovery, neuroprotection, and drug delivery (**Table 2**). While the efficacy of various ultrasound parameters, such as frequency, intensity, duty cycle, and exposure time, has been demonstrated across animal models, there is still a critical gap in understanding the full range of potential risks. Although often described as non-invasive due to the lack of surgical intervention at the clinical trial level, ultrasound is nonetheless capable of producing biologically invasive effects – both therapeutic and harmful. The recent studies reported in **Table 2** have highlighted the therapeutic potential of specific ultrasound protocols, but safety outcomes, although acknowledged, are often not as comprehensively reported.

In earlier feasibility studies, especially those exploring the delivery of ultrasound to uninjured spinal cord tissue, the risk of introducing additional damage was more thoroughly addressed (Ballantine et al., 1956; Bowsher, 1957). However, as we move toward more targeted interventions in injured spinal cords, there is a pressing need for studies that systematically investigate whether certain ultrasound parameters might exacerbate the injury or worsen the condition. Defining the limits of safe ultrasound exposure, particularly in more complex injury models, is essential for ensuring that these treatments do not introduce unintended harm. Importantly, parameters shown to be safe and effective in small animal models may not translate directly to humans, where the anatomical proportions and tissue compositions differ significantly. These differences can result in distinct acoustic interactions and biological responses. Ultimately, understanding the safety thresholds for ultrasound treatment in SCI is just as crucial as optimizing its efficacy, and more studies focusing on these safety aspects will be key to ensuring that ultrasound-based therapies can be safely and effectively translated into clinical practice.

Despite a good body of knowledge detailing the physiological relevance of key ultrasound parameters, it is important to acknowledge the current limitations of defining optimal parameters for human SCI therapy. To date, only one study has explored therapeutic ultrasound in the human spinal cord, where the corticospinal tract of healthy volunteers was targeted (Hou and Lei, 2025). A focused beam with 1 MHz frequency, 10 W/cm² intensity, 30% duty cycle, and 1 kHz PRF was applied. However, these settings were designed for neuromodulation in uninjured tissue and may not translate to injured spinal cords. Optimal parameters are likely to vary depending on the therapeutic goal, e.g., neuromodulation may require higher intensities, while anti-inflammatory effects have been achieved at lower intensities in animal models. Timing is also a critical factor: early post-injury application may mitigate secondary injury cascades, whereas chronic-phase interventions may support neuroplasticity and functional recovery. Ultimately, the lack of human studies in this space underscores the need for further research.

### Choice of transducers

A critical factor in the clinical translation of ultrasound-based therapies for SCI is the appropriate selection and configuration of the ultrasound transducer. Most preclinical studies using ultrasound in SCI have relied on single-element transducers for their relative affordability and simplicity (**Table 2**). These transducers typically have a fixed focus and require manual positioning to align the focal point with the target tissue (Pasquinelli et al., 2020). While this setup works for early proof-of-concept studies, often involving laminectomies, it limits targeting accuracy and reproducibility, especially for dynamic or multi-target interventions.

**Figure 4:**
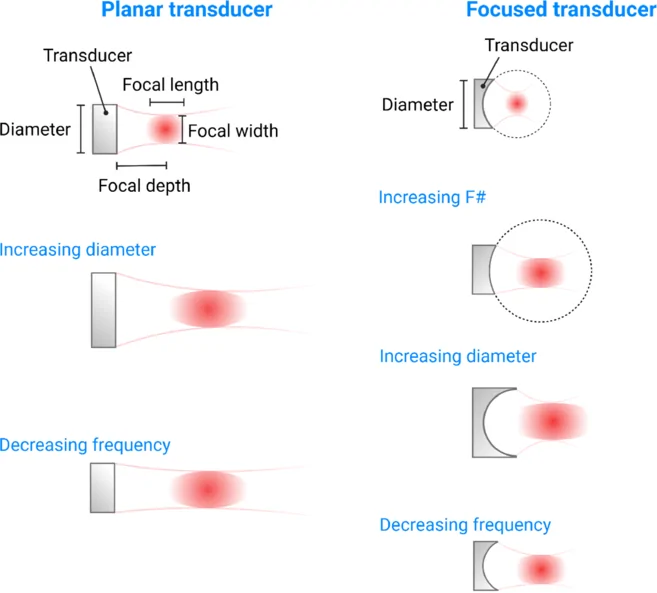
Effects of altering transducer properties on the depth and shape of ultrasound foci. Increasing the diameter of the transducer increases the focal depth, width, and height for both planar and focused transducers. Decreasing the frequency increases the focal depth, width, and height in both transducer types. Increasing the F# has the same effect on focal depth and size as increasing the diameter of the transducer. Created with BioRender.com.

The choice of transducer directly influences the characteristics of the focal zone, namely, the focal depth, width, and length, which ultimately determine where and how acoustic energy is delivered within the spinal cord (**Figure 4**). The focal point must precisely match the anatomical target to ensure therapeutic efficacy while avoiding off-target effects.

Planar and focused transducers offer different focal properties and design considerations. For planar transducers, focal depth can be adjusted by changing either the transducer diameter or the ultrasound frequency. As diameter increases or frequency decreases, focal depth, focal width, and focal length all increase (Javid et al., 2023; Iowa State University, 2025). This means that while planar transducers can be adjusted to reach deeper targets, they may sacrifice spatial precision, especially if the focal zone becomes too wide.

Conversely, focused transducers are designed with a curved surface or an acoustic lens that converges the ultrasound beam to a defined point in space. The radius of curvature determines the focal depth. Simultaneously, the sharpness of focus is often described by the F#, defined as the ratio of the focal depth to the transducer diameter (No author listed, 2025). A lower F# corresponds to a more tightly focused beam with higher energy density and spatial precision. Larger-diameter focused transducers achieve better focusing than smaller ones, enabling more targeted energy delivery at a given frequency.

Another way to adjust the focal depth, width, and length of the wave produced by a focused transducer is by altering the frequency. At higher frequencies, the focal width, length, and depth are lower, while at lower frequencies, the focal width, length, and depth are higher. This means that lower frequencies are more penetrative but will have a larger focus (Lucas et al., 2014; Quarato et al., 2023).

In the context of SCI, where the spinal cord is deeply embedded within the vertebral column and surrounded by heterogeneous tissue structures, these parameters must be carefully tuned. The selected transducer must be capable of reaching the spinal cord through bone and soft tissue while maintaining a focal width narrow enough to be confined to the treatment area (Xu and O’Reilly, 2020). High divergence angles, common in smaller transducers or at lower frequencies, can result in energy dispersion and reduced treatment precision (Imani et al., 2024). Therefore, therapeutic applications targeting the spinal cord, such as neuromodulation, BSCB opening, or localized drug delivery, require transducer designs that balance penetration depth with focal sharpness.

Multi-element array transducers present a solution for overcoming the limitations of single-element transducers. These transducers are made up of many elements that can be arranged as a concentric ring (annular arrays), as a grid, or randomly (Kremkau, 1993; Hand et al., 2009). By carefully controlling the timing of each element’s emission, the ultrasound beam can be steered and focused exactly where it is needed (Dubé, 2004). This is done by calculating the distance between the ultrasound source and the target, along with the speed of sound through the tissue. Using this information, researchers can determine when each transducer should emit its wave so that all waves arrive at the target simultaneously (Hynynen and Jones, 2016). Elements that are further from the target emit their waves earlier, while those closer emit later so that all the waves arrive at the target point at the same time.

This concept can be likened to dropping multiple pebbles into a still pond at carefully chosen positions and time. When timed precisely, the resulting ripples converge at a single point, reinforcing one another to produce a larger wave. Similarly, multi-element transducers emit sound waves with controlled timing, allowing the energy to converge at a defined focal point within the tissue. This coordinated approach enables precise, non-mechanical steering and focusing of the ultrasound beam.

As with single-element transducers, the focal size still depends on determinants such as the transducer diameter, frequency, and F#, but multi-element array transducers require further considerations regarding the steering range. Considering that the spinal cord is a deep structure embedded within heterogeneous structures such as the vertebrae and surrounding soft tissues, multi-element transducers minimize the need for mechanical re-positioning.

### Coupling

In spinal cord ultrasound applications, hair can trap air bubbles that interfere with ultrasound transmission, so the treatment area is typically shaved beforehand to ensure effective coupling. Gel pads and hydrogels are the most commonly used coupling agents, each with distinct advantages. Hydrogels conform well to the skin but can trap small air bubbles that require removal, while gel pads are more stable and less prone to bubbles; however, their thickness (typically around 20 mm) can significantly alter the distance between the transducer and target tissue. Even small variations of a few millimeters can shift the focal point enough to compromise precision, especially in applications where the focal zone is narrow or located close to critical structures. Therefore, careful consideration of the coupling material thickness is essential to maintain accurate targeting.

### Anatomical considerations for transducer positioning and pre-treatment planning

**Figure 5:**
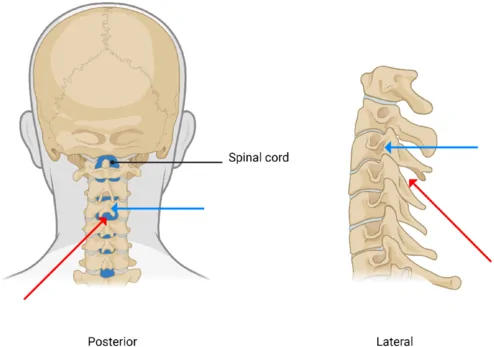
Potential routes of transspinal ultrasound administration to the cervical spinal cord. Blue arrows indicate routes where ultrasound waves pass through bone layer(s), while the red arrows indicate routes where no bone layers are crossed. Created with BioRender.com.

Anatomy plays a crucial role in the application of ultrasound for SCI treatment, as several factors can influence the effectiveness of the therapy. This is an important factor influencing translation, as therapies are first developed in animal models, with vitally different anatomy than the clinical intended use. The anatomical variabilities between individuals and up and down the spinal column mean careful planning and simulations are required to ensure accurate target mapping for ultrasound treatments. As with transcranial applications, the bone-soft tissue boundaries along the path of the ultrasound wave through the spinal cord pose a challenge, as reflection, absorption, and scattering occur due to the impedance mismatch between bone and soft tissue (Jiang et al., 2020; Xu and O’Reilly, 2020). Furthermore, transducer placement sites may be limited depending on the injury level. For example, if the injury level is cervical, the transducers can only be placed posteriorly due to the presence of air-containing structures such as the trachea, which would cause wave reflections. Similarly, posterior positioning is also preferable for application to the thoracic levels, as this minimizes absorption or aberrant reflections of the ultrasound wave by the lungs and ribs. It is also worth noting that due to the anatomy of the spinal cord and vertebrae, ultrasound waves can be delivered between the vertebrae or through bone (**Figure 5**).

Other than the general anatomy and treatment routes, the variation in bone density, particularly in individuals with SCI, is a significant consideration. Bone density can fluctuate due to the progression of SCI, where reduced mobility or altered weight-bearing can lead to bone loss over time (Mun et al., 2024). This reduction in bone density can impact the transmission of ultrasound waves as bone tissues can scatter or absorb the waves. As the density of bone tissue changes, the route of ultrasound delivery and the necessary adjustments to the treatment parameters may also change. This highlights the importance of monitoring bone density in SCI patients and considering these changes when using ultrasound for therapeutic purposes. Personalized treatment will be an important aspect for therapeutic success.

As it stands, there is more clinical experience with transcranial ultrasound stimulation than there is with transspinal ultrasound. Still, there could be some learnings that could be transferred between these technologies about pre-treatment planning. Pre-treatment planning for transcranial ultrasound therapy involves acquiring patient-specific imaging data, typically via computed tomography (CT) or advanced magnetic resonance imaging, to accurately characterize the acoustic properties of the skull (Sigona and Caskey, 2024). These images are processed to generate maps of skull density and speed of sound, which serve as inputs for computational models that simulate ultrasound wave propagation and focusing through the cranial bone. Simulation methods such as the k-space pseudospectral technique or hybrid numerical-analytical approaches are employed to calculate phase corrections and optimize transducer placement and parameters (Wang et al., 2025c). This personalized planning process enhances focal precision while minimizing the risk of off-target heating or cavitation, thereby improving therapeutic efficacy and safety.

Pre-treatment planning methodologies from transcranial ultrasound can be adapted for SCI by accounting for anatomical and acoustic differences between the skull and spinal column. While transcranial planning models skull-bone heterogeneity to correct phase aberrations, SCI planning requires characterization of vertebrae and surrounding tissues using imaging modalities such as CT and magnetic resonance imaging, alongside tailored simulation models due to the complex geometry of the spine. An *ex vivo* study evaluated simulation-based phase corrections for transspine focused ultrasound through human vertebrae, comparing ray acoustics-derived corrections to geometric focusing and a hydrophone-corrected standard by assessing focal shift and similarity metrics (Martin et al., 2024). Results showed significant improvement in targeting accuracy with hydrophone correction, moderate but non-significant improvement with ray acoustics correction and comparable target pressure across methods. These findings suggest that transspinal ultrasound focusing is feasible and, for anatomically favorable targets, may require only minimal corrections. Simulations further revealed that acoustic windows, gaps between bones, can cause unexpected focal shifts, emphasizing the importance of real-time target monitoring in clinical applications.

Encouragingly, recent clinical work has demonstrated the feasibility of targeted spinal cord ultrasound stimulation in healthy human volunteers (Hou and Lei, 2025). This study successfully achieved non-invasive transcutaneous stimulation of the human thoracic spinal cord using LIFU. Feasibility was established by measuring changes in corticospinal excitability: specifically, ultrasound stimulation to the C8 spinal cord segment led to a significant reduction in motor-evoked potential amplitudes elicited by transcranial magnetic stimulation of the motor cortex, indicating a modulatory effect on spinal neural circuits. Notably, this study utilized advanced pre-treatment planning and acoustic simulations to optimize the treatment. The researchers utilized spinal CT data from the VerSe 2020 dataset and performed simulations using k-Plan software to model the spinal cord and surrounding tissues. To minimize interference from dense vertebral structures, the transducer was positioned posteriorly and obliquely, directing the ultrasound beam through the lamina of the sixth cervical vertebra. This strategic placement allowed the beam to bypass the central vertebral body and spinous processes, thus minimizing acoustic distortions such as scattering and refraction.

Using k-Plan, a simulation tool commonly used in transcranial ultrasound applications, the CT images were segmented into vertebral bone, soft tissue, and background based on radiodensity, with the radiodensity values (Hounsfield units) converted to material properties such as density and speed of sound. This enabled the simulation to account for variations in tissue composition across individuals, essential for accurate beam targeting. Acoustic properties, including the absorption coefficient and thermal conductivity, were assigned to each tissue type to refine the simulations further.

Time-domain simulations were then conducted to model the propagation of ultrasound waves through the cervical spine and generate pressure maps. These maps helped confirm effective beam focusing at the C8 spinal cord segment, ensuring that the ultrasound was precisely delivered to the target area. This pre-treatment planning, driven by advanced acoustic simulations, ensured that the ultrasound stimulation was accurately focused on the spinal cord while minimizing off-target effects, thereby optimizing the therapeutic outcome. The study confirmed both safety and the ability to induce motor-evoked potential suppression, providing the first direct evidence of targeted spinal neuromodulation in human subjects. This milestone highlights the critical role of pre-treatment planning and simulations in optimizing ultrasound therapy for SCI and underscores the importance of ongoing research to refine stimulation protocols and delivery systems in the pathological spinal cord.

## Conclusion

Ultrasound therapy holds significant promise as a multifaceted treatment option for SCI, with potential applications ranging from direct regeneration and neuronal stimulation for motor evoked potentials to alleviating spasticity and managing other complications associated with SCI. In preclinical studies, key safety parameters have already been identified. These findings highlight the importance of careful optimization of ultrasound settings to avoid potential adverse effects while maximizing therapeutic benefits.

However, despite these advancements, several key areas require further investigation to fully realize the potential of ultrasound therapy for SCI. First and foremost, future work must focus on the design and development of ultrasound devices specifically tailored for spinal cord stimulation. Current ultrasound devices are generally designed for a wide range of applications and may not be optimized for the unique anatomical and physiological characteristics of the spinal cord. Specialized devices, incorporating innovations in transducer technology, beam shaping, and targeting, are essential for improving the precision and efficacy of spinal cord ultrasound therapy.

Equally critical is the need for a deeper understanding of the relationship between ultrasound dose and biological outcomes, particularly within the context of SCI. While preclinical studies have demonstrated positive effects, there is still a need for more robust data on the dose-response relationship to ensure both the safety and effectiveness of ultrasound stimulation. Defining optimal dosages, such as intensity, frequency, and duration, will be crucial in maximizing therapeutic effects while minimizing risks such as tissue heating or damage. One way forward would be to perform such mechanistic studies with transducers that are implanted into the target tissue in animal models. This would circumvent scattering and other tissue-related variability and yield consistent dosing between experiments, which in turn would greatly facilitate interpretation and mapping of the dose-response. The collected information could subsequently be fed into models, back calculating the non-invasive dosage needed to accomplish the same effect. There is currently a lack of implant technology supporting these types of fundamental studies on ultrasound-tissue interaction.

Another important consideration is the development of standardized protocols for safe administration. Establishing universal guidelines and treatment protocols for spinal cord ultrasound stimulation will ensure consistency in clinical practice, enabling reproducibility and comparability across studies. Treatment planning, informed by advanced simulation models and pre-treatment assessments, should be incorporated into routine clinical workflows to guide the selection of ultrasound parameters tailored to individual patient needs. These protocols will also help standardize safety limits and provide clear recommendations for minimizing adverse effects during treatment.

Taken together, ultrasound therapy represents a promising and versatile therapeutic option for SCI patients. With the refinement of ultrasound technology, the establishment of clear safety guidelines, and a better understanding of the biological mechanisms involved, ultrasound stimulation has the potential to become a treatment option for SCI. Moving forward, collaboration across disciplines and extensive clinical trials will be crucial in translating this technology from experimental settings to everyday clinical practice, ultimately improving the quality of life for SCI patients.

## Data Availability

*Not applicable.*
